# Supplementary material for: Soyasapogenol-B as a Potential Multitarget Therapeutic Agent for Neurodegenerative Disorders: Molecular Docking and Dynamics Study
Source: Entropy (Basel). 2022 Apr 23;24(5):593. doi: 10.3390/e24050593 (PMC9141571; doi:10.3390/e24050593)
Supplement: Supplementary file 1 [file entropy-24-00593-s001.zip › entropy-1561556-supplementary.pdf]

SUPPLIMENTARY FILES

Soyasapogenol-B as a potent multitarget therapeutic agent for neurodegenerative disorders: Molecular docking and dynamics study

Danish Iqbal [1, *], Syed Mohd Danish Rizvi [2,*], Md Tabish Rehman [3], M. Salman Khan [4], Abdulaziz Bin Dukhyil [1], Mohamed F AlAjmi [3], Bader Mohammed Alshehri [1], Saeed Banawas [1,5,6], Qamar Zia [1,5], Mohammed Alsaweed [1], Yahya Madkhali [1], Suliman A. Alsagaby [1], Wael Alturaiki [1]

[1] Department of Medical Laboratory Sciences, College of Applied Medical Sciences, Majmaah University, Majmaah, 11952, Saudi Arabia; da.mohammed@mu.edu.sa (D.I.), a.dukhyil@mu.edu.sa (A.B.D.), w.alturaiki@mu.edu.sa (W.T.), m.alsaweed@mu.edu.sa (M.A.), b.alshehri@mu.edu.sa (B.M.A.), y.madkhali@mu.edu.sa (Y.M.), s.alsaqaby@mu.edu.sa (S.A.A.), qamarzia@mu.edu.sa (Q.Z.)

[2] Department of Pharmaceutics, College of Pharmacy, University of Hail, Hail P.O. Box 2240, Saudi Arabia; sm.danish@uoh.edu.sa (S.M.D.R.)

[3] Department of Pharmacognosy, College of Pharmacy, King Saud University, Riyadh 11451, Saudi Arabia; mrehman@ksu.edu.sa (M.T.R.), malajmii@ksu.edu.sa (M.F.A.)

[4] Clinical Biochemistry & Natural Product research laboratory, Department of Biosciences, Integral University, lucknow-226026, U.P., India; contactskhan@gmail.com (M.S.K.)

[5] Health and Basic Sciences Research Center, Majmaah University, Al Majmaah 15341, Saudi Arabia; s.banawas@mu.edu.sa (S.B.)

[6] Department of Biomedical Sciences, Oregon State University, Corvallis, Oregon 97331, USA.

* Correspondence: da.mohammed@mu.edu.sa; sm.danish@uoh.edu.sa

Supplementary Table S1. Physicochemical properties, percentage absorption and polar surface area of natural BDNF inducer compounds

S. No.	Natural compounds	MW	cLog P**	HBA	HBD	RB	L-V	% GI-A*	TPSA
	Acceptable reference range	<500	≤5	<10	<5	≤10	≤1	≥70%	≤90 Å
1	Soyasapogenol B	458.72	5.56	3	3	1	1	82.59985	60.69
2	Genistein	270.23	1.62	5	3	1	0	71.15935	86.99
3	Huperzine A	242.32	1.29	3	2	0	0	85.0228	55.12
4	Imperatorin	270.28	3.54	4	0	3	0	87.87205	48.57
5	Eugenol	164.2	2.27	2	1	3	0	96.1849	29.46
6	Calycosin	284.26	1.9	5	2	2	0	75.94435	75.99
7	Isorhamnetin	316.26	1.76	7	4	2	0	58.34425	116.45
8	Luteolin	286.23	1.99	6	4	1	0	62.3593	107.22
9	Auraptene	298.38	5.26	3	0	6	1	93.54445	35.53
10	Atranorin	374.34	2.93	8	3	6	0	52.2934	130.36
11	Hydroxytyrosol	154.16	0.8	3	3	2	0	82.59985	60.69
12	Salidroside	300.3	-0.69	7	5	5	0	56.96965	119.61
13	Tanshinone II A	294.34	3.86	3	0	0	0	88.4332	47.28
14	Withanolide A	470.6	2.56	6	2	2	0	67.0834	96.36
15	Caffeine	194.19	-0.178	6	0	0	1	83.5786	58.44
16	L-theanine	174.19	-2.99	5	3	5	0	68.7973	92.42
17	Catechin	290.27	1.5	6	5	1	0	60.9847	110.38
18	Honokiol	266.33	4.68	2	2	5	0	91.3999	40.46
19	Magnolol	266.33	4.68	2	2	5	0	91.3999	40.46
20	Chlorogenic acid	354.31	-0.76	9	6	5	1	37.33375	164.75
21	Alpinetin	270.28	2.77	4	1	2	0	84.7444	55.76
22	Fisetin	286.23	1.83	6	4	1	0	62.3593	107.22
23	Scopoletin	192.17	1.08	4	1	1	0	84.7444	55.76
24	Apigenin	270.23	2.33	5	3	1	0	71.15935	86.99
25	Silibinin	482.44	2.12	10	5	4	0	41.5141	155.14
26	Nodakenin	408.4	0.024	9	4	4	0	50.31415	134.91
27	Albiflorin	480.46	-1.35	11	5	7	1	34.08865	172.21
28	Picrotoxinin	292.28	-0.68	6	1	1	0	71.8684	85.36
29	Kainic acid	213.23	-2.02	5	3	4	0	71.31595	86.63
30	Epigallocatechin-3-O-gallate	442.37	2.4	10	7	4	1	31.9441	177.14
31	Hericene A Chemspider	556.82	11.62	5	1	24	3	77.31895	72.83
32	Physodic acid	470.51	6.07	8	3	11	2	52.2934	130.36
33	Perlatolic acid	444.52	6.05	7	3	13	2	59.71885	113.29
34	Ginsenosides Rg1 Chemspider	801.02	2.117	14	10	10	3	4.9393	239.22
35	Ginsenosides Rb1 Chemspider	1109.3	-7.048	23	15	16	4	-55.12115	377.29
36	Salvianolic acid B Chemspider	718.61	2.91	16	9	14	4	-11.9474	278.04
37	Bacoside A Chemspider	927.17	1.28	16	8	10	3	6.4792	235.68
38	Crocin	976.96	-1.9	24	14	20	4	-61.172	391.2
39	Fucoxanthin	658.91	9.5	6	2	12	3	67.0834	96.36
40	Hyperforin	536.79	9.81	4	1	11	3	77.9236	71.44
41	Hypericin	504.44	4.8	8	6	0	2	41.3488	155.52
42	Aconitine	645.74	0.54	12	3	11	3	42.24925	153.45
43	Hypaconitine	615.71	0.99	11	2	10	2	51.0493	133.22
44	Mesaconitine	631.71	0.138	12	3	10	2	42.24925	153.45

**Logarithm of compound partition coefficient between *n*-octanol and water

Molecular weight (MW), number of hydrogen bond donors (HBD), number of hydrogen bond acceptors (HBA), number of rotatable bonds (RB), cLogP value (clogP), topological polar surface area (TPSA), Lipinski's rule violation (L-V), and human gastrointestinal absorption (GI-A).

Supplementary Table S2. Toxicity Potential of Natural BDNF inducer compounds

S. No.	Natural compounds	Mutagenic	Tumorigenic	Reproductive Effect	Irritant
1	Soyasapogenol B	None	None	None	None
2	Genistein	High	High	High	None
3	Huperzine A	None	None	None	None
4	Imperatorin	High	None	Low	None
5	Eugenol	High	High	None	High
6	Calycosin	None	None	None	None
7	Isorhamnetin	High	None	None	None
8	Luteolin	None	None	None	None
9	Auraptene	None	None	None	High
10	Atranorin	None	None	None	None
11	Hydroxytyrosol	None	None	None	None
12	Salidroside	None	None	None	None
13	Tanshinone II A	None	None	High	None
14	Withanolide A	None	Low	Low	Low
15	Caffeine	High	High	High	None
16	L-theanine	None	None	None	None
17	Catechin	None	None	None	None
18	Honokiol	None	High	None	High
19	Magnolol	None	High	None	High
20	Chlorogenic acid	None	None	None	None
21	Alpinetin	None	None	None	None
22	Fisetin	High	None	None	None
23	Scopoletin	None	None	Low	None
24	Apigenin	High	None	None	None
25	Silibinin	None	None	None	None
26	Nodakenin	None	None	None	None
27	Albiflorin	None	None	Low	High
28	Picrotoxinin	None	None	None	High
29	Kainic acid	None	None	Low	None
30	Epigallocatechin-3-O-gallate	None	None	None	None
